# Assessment of 24-h moving average PM_2*.*5_ concentrations in Bangkok, Thailand against WHO guidelines

**DOI:** 10.1186/s42834-023-00165-y

**Published:** 2023-01-24

**Authors:** Sirapong Sooktawee, Suwimon Kanchanasuta, Natthaya Bunplod

**Affiliations:** 1 Environmental Research and Training Center, Ministry of Natural Resources and Environment, Pathum Thani, 12120 Thailand; 2 Faculty of Public Health, Mahidol University, Bangkok, 10400 Thailand; 3 Center of Excellence on Environmental Health and Toxicology, Bangkok, 10400 Thailand

**Keywords:** Bangkok, PM_2*.*5_, Euclidean distance, Cross-correlation, Moving average

## Abstract

Particulate matter less than 2.5 μm in diameter (PM_2.5_) pollution has gained more concern, as can be seen from the World Health Organization (WHO) revised air quality guideline (AQG) value. To compare with the 24-h AQG value, the 24-h mean (daily average) of hourly monitoring data has been widely used, but the nature of data in hourly variation has been lost. The 24-h moving average can be an alternative approach to preserving hourly data behavior, while various positions of the hour (leftmost, center, or rightmost hour) can be used to store the moving average. In this study, similarity testing by cross-correlation and Euclidean distance was performed to present a suitable 24-h moving average time series for hourly data, and then presented proportions of PM_2.5_ concentrations within each interim target and AQG ranges. The PM_2.5_ time series of the leftmost and rightmost 24-h moving average revealed lags of − 13 to − 10 h and 10 to 13 h for the observed hourly data, respectively. The lags of the center 24-h moving average time series were of − 2 to 1 h, which showed more similar events to the hourly PM_2.5_ fluctuation than the leftmost and rightmost time series. The center 24-h moving average concentrations measured in Bangkok were categorized into interim target and AQG ranges. The results revealed that the proportion of concentrations lower than AQG during nighttime and daytime was approximately 40 and 28%, respectively. However, the results given from the leftmost and rightmost time series showed time shifts and differences in diurnal variation caused by the position used to store the average value. To keep hourly PM_2.5_ variation that can be compared with the 24-h WHO guidelines, this should be performed with careful and precautionary consideration of misinterpretation over a time scale, especially for implementing countermeasures and policies.

**Supplementary Information:**

The online version contains supplementary material available at 10.1186/s42834-023-00165-y.

## Introduction

Air pollution, especially that caused by particulate matter less than 2.5 μm in diameter (PM_2*.*5_), remains a concern in many countries such as Australia, Brazil, China, India, Iran, Japan, Portugal, Thailand, and the United States [1–8]. Addressing PM_2*.*5_ pollution is essential for sustainable development. Its annual mean levels have been included as a target to be met by 2030 under the United Nations Sustainable Development Goals (SDGs), thereby reducing the adverse per capita environmental impact in cities [9]. PM_2*.*5_ pollution has affected health in many areas [10], and it includes carcinogenic substances such as polycyclic aromatic hydrocarbons (PAHs), whose concentration has been reported to be higher in urban areas than in semiurban and rural areas [11]. The WHO revised air quality guidelines in 2021 to challenge the global community to enhance air quality and reduce the health burden. Compared with the 2006 guidelines, in the 2021 guidelines, the recommended 24-h mean and annual mean PM_2*.*5_ values were revised from 25 to 15 μg m^− 3^ and from 10 to 5 μg m^− 3^, respectively [12, 13].

A previous study compared the air quality guideline (AQG) value for a 24-h mean PM_2*.*5_ concentration with the actual ambient PM_2*.*5_ levels in 45 megacities in the world and found none to meet even the 2006 AQG value (25 μg m^− 3^) [14]. A study from rural South India reported high PM_2*.*5_ concentrations during winter, with daily average concentrations exceeding the former 24-h AQG most (76–98%) of days, and PM_2*.*5_ pollution episodes existing 7–19% of the total hours [15]. The coastal area of Pattaya, Thailand showed a maximum of 24-h moving average PM_2*.*5_ concentrations close to the former 24-h AQG value [16]. In Chile, the PM_2*.*5_ level was defined as “good” when the 24-h moving average concentration was < 50 μg m^− 3^ [17], meeting the PM_2*.*5_ Interim Target-2 of both 2006 and 2021 WHO guidelines [12, 13]. The study used a neural network for PM_2*.*5_ forecasting in Chile and successfully provide forecast hourly concentration; the 24-h moving average maxima between the observed and forecasted data were comparable [17]. The National Environmental Agency of Singapore warned residents regarding the 24-h moving average PM_2*.*5_ concentration of 310 μg m^− 3^ on June 20, 2013. The maximum 24-h moving average PM_2*.*5_ concentration decreased to 302 μg m^− 3^ and greatly increased to 382 μg m^− 3^ on June 22, 2013, and June 22, 2013, respectively. This excessive PM_2*.*5_ concentration was reported to affect the residents [18]. These examples demonstrated the various applications of methods used to calculate and compare the 24-h average AQG, including the daily average and 24-h moving average. Many countries have adopted the U.S. National Ambient Air Quality Standards (NAAQS) for 24-h PM_2*.*5_ concentrations.

PM_2*.*5_ levels in megacities are monitored using different measurement techniques including gravimetric, beta attenuation, tapered element oscillating microbalance (TEOM) and TEOM fitted with a filter dynamics measurement system [14]. The gravimetric method provides the average concentration representing for sampling period, i.e., 24 h, whereas continuous PM_2*.*5_ measurement methods, such as beta attenuation and TEOM can provide continuous hourly concentrations. To calculate the 24-h concentration average of PM_2*.*5_ from continuous monitoring equipment, the US EPA requires the data of at least 75% of the 24-h period used in calculating. The 24-h average is stored in the first hour, which is 0:00 [19]. Therefore, averaging every block of 24-h data points (0:00-23:00) through the end of the time series yields a new time series of daily average concentrations. In addition to storing the average value at the start, it can be stored in the middle or at the end [20]. The disadvantages of using the simple block average method to generate daily time series is the loss of intraday concentration fluctuation. The moving average method is the simple block average computation over 24 h, including 0:00 to 23:00, to obtain a mean value; then, the average value for the subsequent period (1:00-0:00) is calculated. The time series results provided using the 24-h moving average method can reveal variations in hourly PM_2*.*5_ concentrations and be evaluated using the WHO 24-h AQG and NAAQS. The moving average technique is often used to smooth data and depict trends. The trailing moving average method can be used to predict future values, whereas the central moving average technique is perhaps more appropriate for representing the actual fluctuation in time series [20]. However, examining the three recorded positions (left, center and right) of the 24-h moving average is crucial to determine whether one of them represents time series fluctuation and captures the high concentration event of hourly PM_2*.*5_ concentration better than the others.

The similarity can indicate the analogous characteristics between two time series data. Euclidean distance is a widely used time series similarity measures [21–23]. Another index is the cross-correlation function (CCF) or Pearson’s correlation function [23]. The CCF evaluates similarity in time series fluctuation shift (shape), whereas Euclidean distance evaluates similarity tests in terms of different distances between two time series (magnitude). The correlation coefficient was used in a study to measure the similarity of stock prices, air temperatures, sea temperatures, wind speeds and electroencephalograms [22]. Euclidean distance was employed to compare wind speed variation between many monitoring sites for the same wind direction [24]. A study analyzing air quality data used the square of Euclidean distance and correlation to compare the samples and the reference [25]. Given that variations in the three moving average time series (left, center, or right) differ from the hourly observed data behavior, when comparing 24-h moving average PM_2*.*5_ concentration data and the AQG or NAAQS, it should be examined which moving average series is the most similar to the hourly time series and captured hourly fluctuation.

In this study, we performed similarity testing using cross-correlation and Euclidean distance to provide an acceptable 24-h period and then compared the three types of the 24-h moving average PM_2.5_ concentration data with the observed hourly data and the AQG or NAAQS to offer an acceptable 24-h period. Our study is expected to maintain the data characteristics, and proposes arguments for or against the choice of data analysis for further studies on air quality and others.

## Methods

### Air quality data

**Fig. 1:**
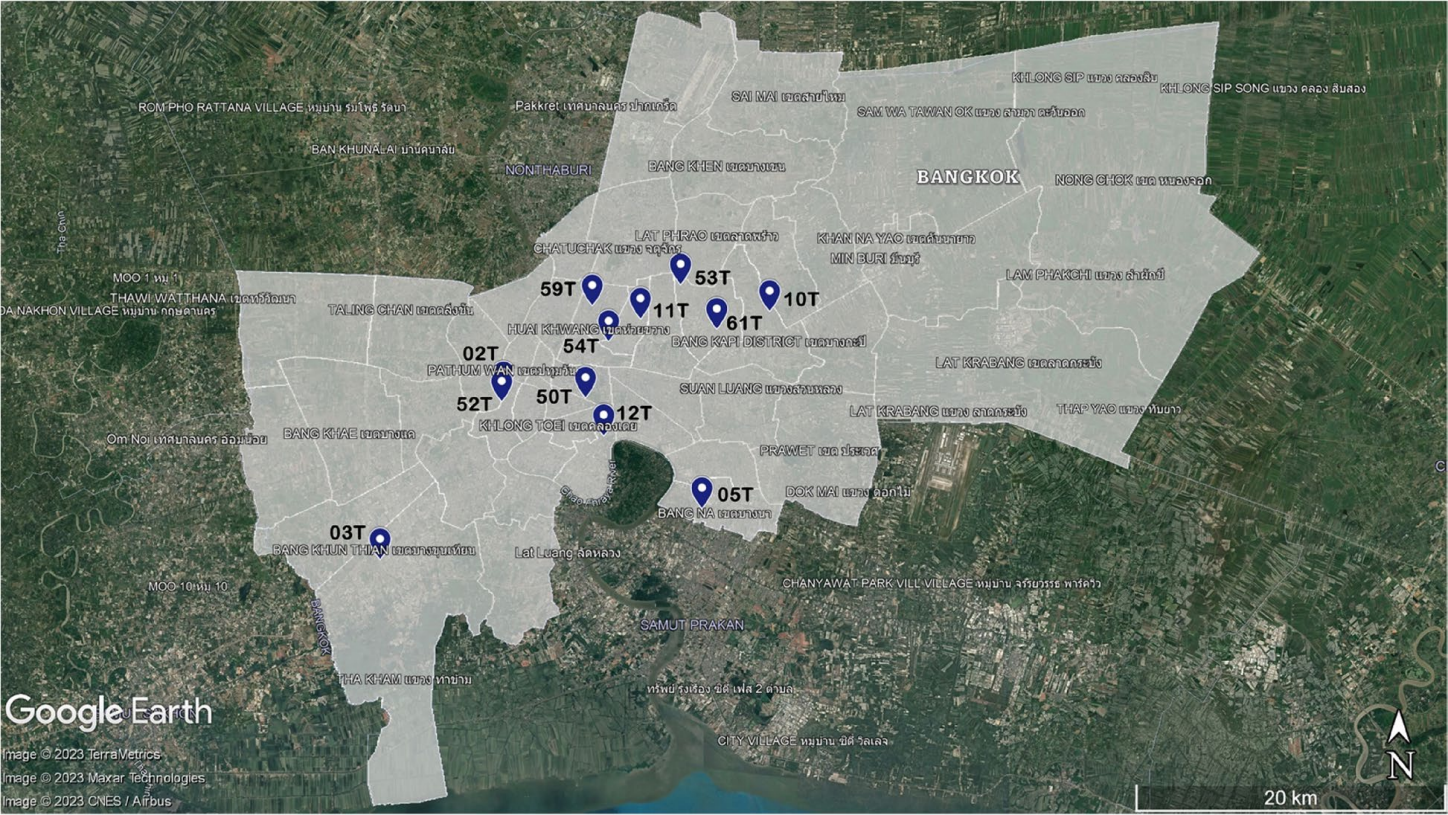
Locations of air quality monitoring sites

We obtained hourly PM_2*.*5_ observed data from 12 air quality monitoring stations at various places in Bangkok, Thailand; the data sets were provided by Thailand’s Pollution Control Department (PCD). Of the 12 stations, five are located within 5 m of the road (the PCD classifies them as “roadside stations”), and the other seven are located in residential areas (Table 1 and Fig. 1). The continuous PM_2*.*5_ monitoring equipment used met the methods recognized by the PCD and also met the standards of the US EPA.

**Table 1:** Description of monitoring data

Station ID	Period	Description	Lat	Lon
02 T	August 18, 2019 to December 31, 2020	Residential area	13.7328 N	100.4877 E
03 T	October 17, 2018 to December 31, 2020	Roadside	13.6365 N	100.4143 E
05 T	January 1, 2018 to December 31, 2020	Residential area	13.6662 N	100.6057 E
10 T	October 17, 2018 to December 31, 2020	Residential area	13.7799 N	100.6460 E
11 T	October 17, 2018 to December 31, 2020	Residential area	13.7755 N	100.5692 E
12 T	August 18, 2019 to December 31, 2020	Residential area	13.7081 N	100.5473 E
50 T	January 1, 2018 to December 31, 2020	Roadside	13.7299 N	100.5365 E
52 T	January 1, 2018 to December 31, 2020	Roadside	13.7276 N	100.4866 E
53 T	January 1, 2018 to December 31, 2020	Roadside	13.7954 N	100.5930 E
54 T	25 January 2018 to December 31, 2020	Roadside	13.7925 N	100.5502 E
59 T	January 1, 2018 to December 31, 2020	Residential area	13.7832 N	100.5405 E
61 T	January 1, 2018 to December 31, 2020	Residential area	13.7697 N	100.6146 E

### Statistical analysis

To compare the observed PM_2*.*5_ data with the 24-h average AQG value, the hourly PM_2*.*5_ concentrations were computed as the 24-h average using the moving average method. This moving average was stored in the first, middle, or last hour (or leftmost, center, and rightmost hour) by using the OpenAir package for air quality analysis, and the similarities between the three types of values were compared. The CCF and Euclidean distance were used to compare the leftmost, center, and rightmost moving average PM_2*.*5_ time series with hourly PM_2*.*5_ time series. Similarities in terms of fluctuation shape and distance between the two time series were analyzed using CCF and Euclidean distance, respectively.

Several studies have used CCF for various reasons—for example, to examine the association between confirmed cases of COVID-19 and meteorologic variation [26] to evaluate the relationship between the El Nino-Southern Oscillation variability represented by the Southern Oscillation Index and the associated time series of the number of new fish [27], and to investigate the lead–lag relationship between the two time series at different time points; CCF can also be used to determine the optimal time shift between the two time series [27, 28]. The correlation coefficients of 1 and − 1 indicate perfect relationships in the same and opposite directions, respectively, with other positive or negative values implying the following: no relationship (0), almost negligible relationship (< 0.2), small relationship (0.2–0.4), substantial relationship (0.4–0.7), marked relationship (0.7–0.9) and very dependable relationship (0.9–1.0), respectively [29]. The CCF described by Chatfield and Xing [28] was calculated as

1 $${\gamma}_{XY}(k)=\frac{C_{XY}(k)}{S_X{S}_Y}$$

where *γ*_*XY*_(*k*) is cross-correlation coefficient at lag *k*. *C*_*XY*_ is cross-covariance function, *S*_*X*_ is sample standard deviations of time series *X*, and *S*_*Y*_ is sample standard deviations of time series *Y*. The equations used to determine the cross-covariance function are

2 $${C}_{XY}=\sum\nolimits_{i=1}^{N-k}\left({x}_t-\overline{x}\right)\left({y}_{t+k}-\overline{y}\right)/N,\,N=0,\,1,\,\dots,\, N-1$$

and

3 $${C}_{XY}=\sum\nolimits_{i=1-k}^N\left({x}_t-\overline{x}\right)\left({y}_{t+k}-\overline{y}\right)/N,\,N=-1,\,-2,\,\dots, \,-\left(N-1\right)$$

where the lag time point is *k*, which is usually much less than the number of time points along the sample time series (*N*).

When the two data sets have very positive dependable relationships, their temporal variations are similar. We examined the relationships between the 24-h moving average PM_2*.*5_ time series and its hourly time series to reveal the lead–lag correlations of 72 time points (h). A time point showing the highest positive correlation coefficient, indicates the best shape similarity of both time series at this time point. A good representation of the 24-h time series for the hourly time series would have a high correlation coefficient and a short lead or lag time length of the time point. The highest correlation presenting at a time point of zero indicates no lead or lag time. This similarity is shape-preserving, but has different magnitudes (vertical shifts) between two time series. Comparison between the two time series based on the concept of distance measures can be performed using time series similarity measures, including Euclidean distance and dynamic time warping (DTW) [21, 23, 30–32]. Euclidean distance is based on the point-to-point measurement concept whereas DTW is based on the concept of one-to-many to obtain minimum distance. Both concepts are visualized in graphic form in the studies of Serra and Arcos [32] and Cassisi et al. [23]. We used the point-to-point distance concept because we considered the coincident events between the 24-h moving average PM_2*.*5_ and hourly PM_2*.*5_ time series. The calculation of similarity represented by the Euclidean distance [21, 33] can be determined using the following equation:

4 $${E}_D=\sqrt{\sum\nolimits_{i=1}^N{\left({x}_i-{y}_i\right)}^2}$$

Less distance resulting in less vertical shift is more similar between both time series. Therefore, Euclidean distance and CCF analyses were calculated to evaluate the three types of the 24-h moving average. Next, we analyzed the 24-h moving average PM_2.5_ time series against the 24-h PM_2.5_ average values suggested by the WHO AQG. The 24-h moving average PM_2.5_ data were binned into each hour: 0:00, 1:00, ..., 23:00. Frequencies of concentrations falling in AQG, interim target (IT) 1, 2, 3, 4, and above were calculated for each hour as follows:

5 $${F}_{Th,h}=\frac{n_{Th,h}}{N_h}$$

where *F*_*Th*, *h*_ is the frequency of concentrations falling in each threshold (*Th*) ranges (AQG: ≤ 15 μg m^− 3^, IT4: 15–25 μg m^− 3^, IT3: 25–37.5 μg m^− 3^, IT2: 37.5–50 μg m^− 3^, IT1: 50–75 μg m^− 3^, and > 75 μg m^− 3^) of an hour (*h*). *N* is the total number of concentration values in an hour (*h*), and *n* is the number of concentration values in the threshold (*Th*) in an hour (*h*). The summation of *F*_*Th*, *h*_ on a particular hour equals 100. Visualization all of *F*_*Th*, *h*_ reveals the diurnal variation of each contribution of AQG and ITs.

## Results and discussion

### Investigation of representativeness on method recording moving average value

**Fig. 2:**
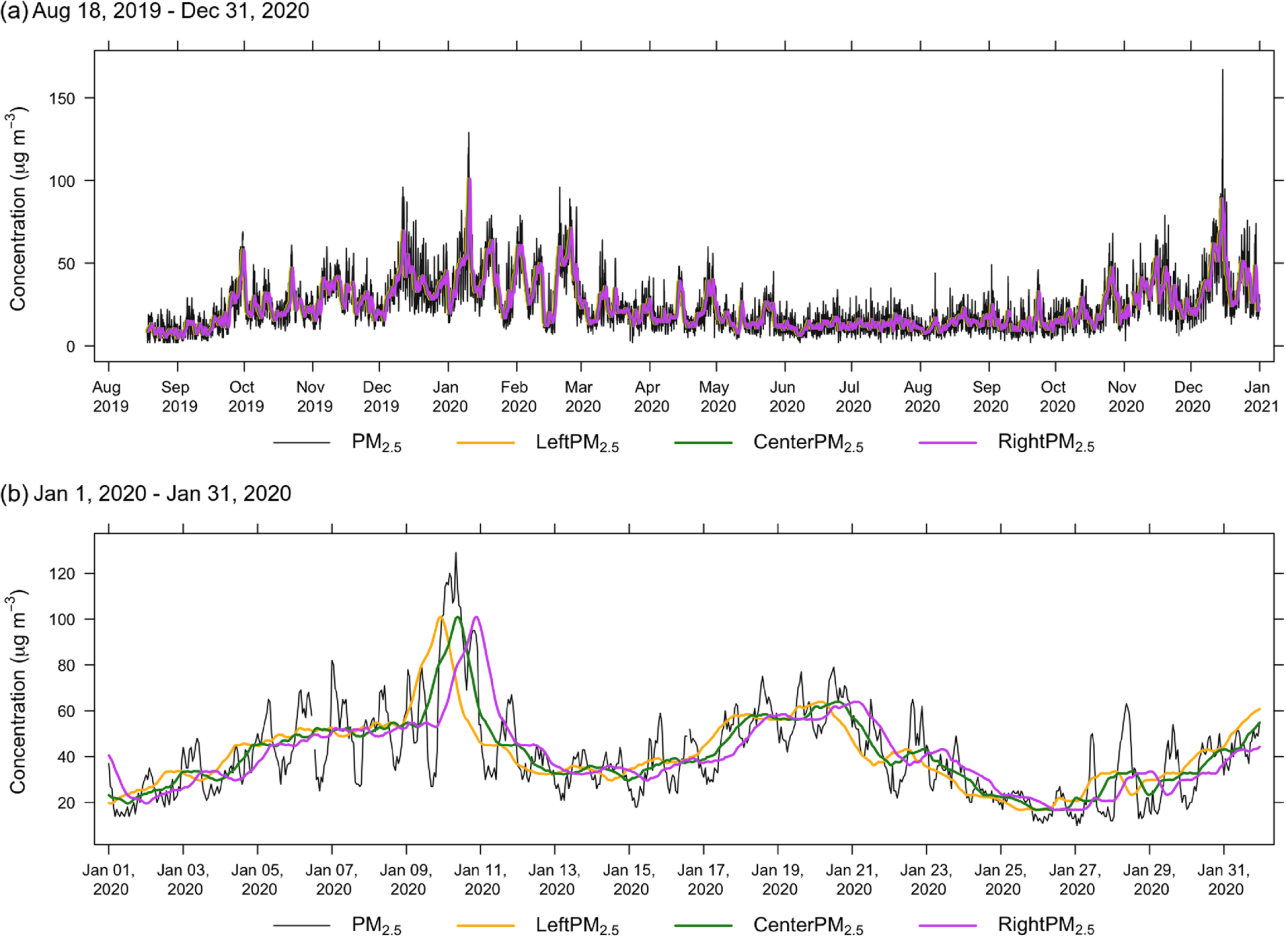
Time series variation of hourly PM_2.5_ concentrations (black line) measured at station 02 T and its 24-h average PM_2.5_ concentrations recorded at leftmost (yellow line), center (green line), and rightmost (purple line), respectively

**Fig. 3:**
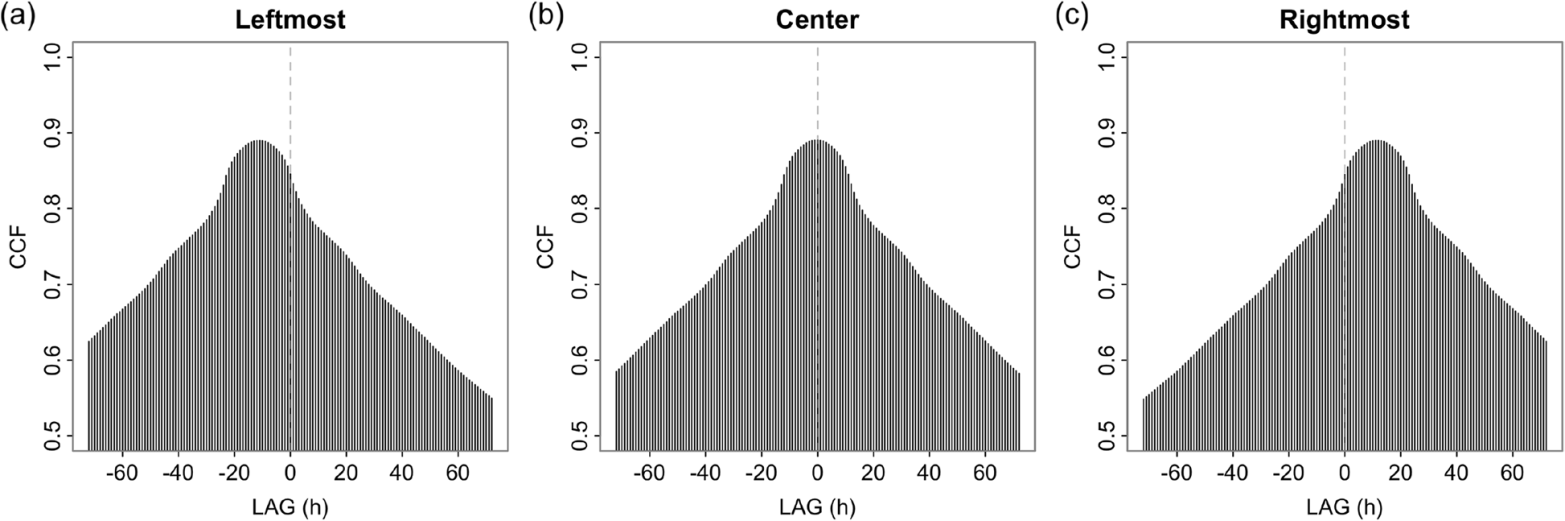
Correlograms of cross-correlation values between hourly PM_2.5_ concentrations measured at station 02 T and its 24-h average PM_2.5_ concentrations recorded at leftmost, center, and rightmost, respectively

The three types of 24-h moving average recording method resulted in the time shifting of high concentration peaks to the peak of hourly time series data (Fig. 2). For a long time series period, the difference between line graphs of three 24-h moving average PM_2*.*5_ concentration time series and hourly monitoring data is difficult to investigate. What type of recording position of 24-h moving time series is appropriate to capture fluctuations of hourly PM_2*.*5_ time series? Examinations using correlation analysis between hourly time series data and each 24-h moving average time series (leftmost, center rightmost) were performed by the CCF. This provides a measure of the similarity between the two time series when a curve shift is found to the original time series. Correlations between both signals at lead/lag of 72 time points (time steps) reveal their temporal relationship. A high CCF value indicates a strong relationship representing high similarity [34]. In this case, three types of 24-h average fluctuations may signal a time shift to the hourly signal (Fig. 2a). A shorter period shift between a 24-h average data set and the hourly data set means a greater possibility of representing the hourly data set. Figure 3 presents the results given by the CCF analysis for station 02 T. The highest correlation coefficient is 0.89 at lag times from − 10 to − 12 h meaning the peak of the leftmost 24-h average PM_2*.*5_ time series occurring before the peak of the hourly PM_2*.*5_ time series is approximately 10 to 12 h. For the 24-h moving average recording at the center, the highest correlation coefficient was 0.89 at a lag time from − 1 to 0 h revealing coincident peaks occurring in both time series (Fig. 3b). The last one had the highest correlation (0.89) of the rightmost 24-h moving average of PM_2*.*5_ time series to hourly time series at lags from 10 to 13 h. The high PM_2*.*5_ peak of the 24-h time series arrived later than the peak of the hourly PM_2*.*5_ time series at approximately 10 − 13 h (Fig. 3c). The results provided by the CCF analysis for other monitoring stations exhibit similar results as shown in the Supplementary Materials. A summary of the lag times and correlation coefficients of all stations in this study is presented in Table 2. Station 11 T exhibited the highest correlation of 0.893 for the center and rightmost averages with lags from − 1 to 0 h and 11 h, respectively. The lowest correlation coefficient presented at station 03 T, with 0.819 for the leftmost and rightmost 24-h moving averages, with time lags from − 12 to − 10 h and 10 to 13 h, respectively. Overall, they presented highly marked relationships. For lead and lag time between them, the leftmost, center, and rightmost 24-h moving average are lags from − 13 to − 10 h, − 2 to 1 h, and 10 to 13 h, respectively. The center 24-h moving average produces time series peaks coinciding with the high concentration peaks of the hourly time series more than the others. Figure 2b presents the time variation of PM_2*.*5_ for hourly, leftmost, center, and rightmost 24-h moving average time series from January 1 to 31, 2020. The 24-h moving average time series exhibited less fluctuation than that of hourly time series data because the moving average method smoothed the data but still captures concentration fluctuation.

**Table 2:** Highest correlation and its corresponding lag (h) of all stations

Station ID	Rightmost		Leftmost		Center	
	Lag	R	Lag	R	Lag	R
02 T	10 to 13	0.890	−12 to − 10	0.890	− 1 to 0	0.891
03 T	10 to 13	0.819	−12 to − 10	0.819	− 1 to 0	0.820
05 T	11 to 13	0.881	−12 to − 10	0.881	0	0.882
10 T	10 to 13	0.868	−12 to − 10	0.868	− 1 to 0	0.869
11 T	11	0.893	−13 to − 10	0.892	− 1 to 0	0.893
12 T	11	0.861	−13 to − 10	0.860	−2 to 1	0.861
50 T	11 to 12	0.880	−12 to − 10	0.880	− 2 to 1	0.880
52 T	11 to 12	0.891	−12 to − 11	0.891	−2 to 1	0.891
53 T	10 to 13	0.872	−12 to −10	0.872	−2 to 1	0.872
54 T	10 to 12	0.839	−13 to −10	0.839	−1 to 0	0.84
59 T	10 to 12	0.858	−13 to −11	0.858	−1	0.859
61 T	10 to 12	0.885	−13 to −10	0.885	−1 to 0	0.886

**Fig. 4:**
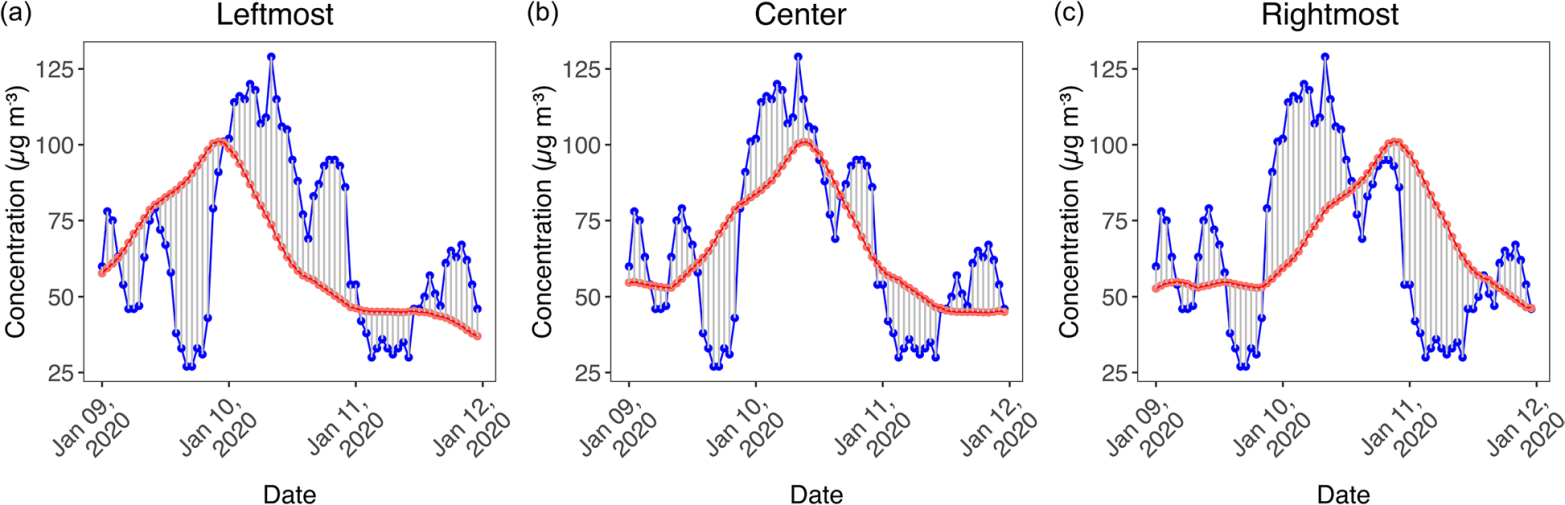
Distance between hourly PM_2.5_ concentrations measured at station 02 T and its 24-h average PM_2.5_ concentrations recorded at leftmost, center, and rightmost, respectively. Blue color represents hourly time series data, red color represents 24-h moving average time series data, and gray color is point to point distance

The 24-h moving averages tended to change in hourly time series. Variations of leftmost, center, and rightmost 24-h moving averages PM_2*.*5_ concentrations revealed associations with hourly PM_2*.*5_ variation that occurs before, coincident, and after to hourly variations, respectively, resulting from the CCF analysis. On January 8–12, 2020, the leftmost 24-h moving average time series started January 9, 2020 to the highest concentration at 23:00, whereas the hourly concentration time series presented the highest concentration January 10, 2020 at 8:00, meaning that the leftmost 24-h moving average time series indicated the peak event before it occurred (Fig. 4a). The 24-h moving average concentrations recorded at the center was quite constant from 0.00 to 7:00 January 9, 2020, and thereafter, concentration continued rising to a peak January 10, 2020 7:00–9:00, which was closest to a peak event of the hourly time series (Fig. 4b). The 24-h moving averages recorded at the rightmost maintained a quite constant low concentration from 0:00–20:00 January 9, 2020, and the highest concentration was observed at 21:00 January 10, 2020 occurring later than the highest concentration of hourly time series January 10, 2020 at 8:00 (Fig. 4c). We conclude that the 24-h moving average PM_2*.*5_ concentrations recorded at the center were more similar to the fluctuation of the hourly PM_2*.*5_ time series than the others. This constitutes a similarity of 24-h moving average time series to hourly time series in terms of shape fluctuation.

Another measure is the similarity in terms of distance. Euclidean distance has been widely used to examine similarity and has been used to describe the terms of distance between two time series. The distance is determined by taking the square root of the sum of the squared differences between points to points of the corresponding time series. The concept of point-to-point distance is illustrated in Fig. 4. The calculated distance between the leftmost 24-h average PM_2*.*5_ time series and the hourly PM_2*.*5_ time series resulting from Eq. (4) is 912 μg m^− 3^. The distances of the center 24-h average PM_2*.*5_ time series and the leftmost 24-h average PM_2*.*5_ time series to the hourly PM_2*.*5_ time series are 777 and 911 μg m^− 3^, respectively. Euclidean distance presented in Eq. (4) is related to the summation of point-to-point distance along the time series. A related study considered the number of points in determining the Euclidean distance between point to origin through the whole data length by dividing the summation by the number of points [30]. Thus, we calculated the square root of the sum of squared distances (Euclidean distance) divided by the number of points ($\sqrt{E_D^2/N}$) and hereafter referred to the averaged Euclidean distance. It presents the distance in terms of the average distance between the two time series. The averaged Euclidean distances between the leftmost, center, and rightmost 24-h average PM_2*.*5_ time series and hourly time series were 8.4, 7.1, and 8.4 μg m^− 3^, respectively. The center 24-h average PM_2*.*5_ time series showed the smallest value. According to the Euclidean distance of 0 representing the perfect similarity in terms of distance, increasing the Euclidean distance is related to reducing the similarity. Therefore, the center 24-h average PM_2*.*5_ time series was more similar to the hourly PM_2*.*5_ time series than to the rest of the time series and reduced the similarity. Therefore, the center 24-h average PM_2*.*5_ time series was more similar to the hourly PM_2*.*5_ time series than to the rest of the time series. We also calculated the mean value and the mean absolute value of point-to-point distances along the time series. The mean values of the leftmost, center, and rightmost 24-h average PM_2*.*5_ time series to the hourly time series were zero because of moving average smoothing hourly data and canceling the upper and lower residuals. The absolute mean values of distances were 5.8, 0, and 5.9 μg m^− 3^ for the leftmost, center, and rightmost 24-h average PM_2*.*5_ time series, respectively. The reason the three mean absolute values were not zero was the mean values, because the absolute mean value of distance does not account for the positive and negative directions of each distance. The mean value and mean absolute value of distances are less suitable for describing the similarity in terms of distance than Euclidean distance.

### State of PM_2.5_ level associated with WHO guidelines

**Fig. 5:**
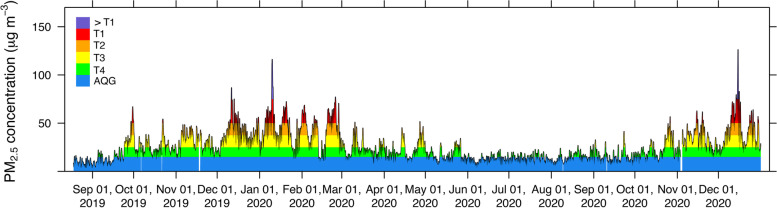
Time series of 24-h average (center) PM_2.5_ concentration measured at station 02 T

In 2021, WHO updated the air quality guidelines, with PM_2*.*5_ level classification of 24-h average concentration as five levels. The 1st, 2nd, 3rd, and 4th interim targets and the guideline values were 75, 50, 37.5, 25, and 15 μg m^− 3^, respectively [13]. Thailand has responded to a new version of the guidelines by revising the standard value (annual average) of PM_2*.*5_ to 5 μg m^− 3^. For the 24-h average standard value, the update is on a process revising the value of 50 to be 37.5 μg m^− 3^. The improved standard of 24-h average value would affect the state of PM_2*.*5_ level. The 24-h average concentrations of the station 02 T were plotted by shading with PM_2*.*5_ level classification of WHO guidelines (Fig. 5). Concentration levels during the red shade and above were greater than the interim target 2 (50 μg m^− 3^), namely, the previous Thai standard value. The high concentration periods over 50 μg m^− 3^ were late September 2019 to March 2020 and October 2020 to December 2020 (end of data) because atmospheric conditions do not favor pollutant dispersion. These periods occurred during the transition season (summer to winter monsoon) and winter. The climatic conditions that govern Thailand and neighboring countries during winter is that the winter monsoon decreases temperature during this period [35]. If PM_2*.*5_ emissions in an area are constant, as emissions from transportation and industrial sectors are quite stable in terms of time variation throughout the year, the mass of PM_2*.*5_ would also be constant. The factor related to change in concentration is the volume of air, which is the area at ground level multiplied by the height. The area does not change whereas the planetary boundary layer (PBL) height can vary. In the Northern Hemisphere, the variability of PBL indicated that PBL height decreased during winter and increased during summer [36]. Therefore, reducing PBL height during winter reduced the air volume, which constitutes an essential factor in enhancing PM_2*.*5_ concentration in the atmosphere, even when no emission increases. A one-half decrease in PBL height corresponds to a one-half decrease in air volume doubling the increasing concentration.

Another factor is PM_2*.*5_ emissions during winter, mainly due to traffic, biomass and open burning. The number of fire hotspots used to represent open burning was considerably greater in winter than that in the other seasons. Because the increase in registered vehicles in Bangkok and fuel consumption varies less than the intra-annual variation in biomass and open-burning emissions, the transport sector emission may be more or less constant throughout the year. However, the time series of PM_2*.*5_ illustrated in Fig. 5 exhibits high concentrations throughout the year over the interim target 2 level (25 μg m^− 3^). Overall, PBL height reduction and emissions from open burning were factors that enhanced the severity of PM_2*.*5_ concentration during the winter. With them, the PM_2*.*5_ level remains above the threshold, the interim target 2 (25 μg m^− 3^), and some would exceed the interim target 3 (37.5 μg m^− 3^) value. Accordingly, the 24-h average PM_2*.*5_ standard level of Thailand was strengthened from 50 μg m^− 3^ (interim target 2) to 37.5 μg m^− 3^ (interim target 3). Achieving the new standards is possible by reducing emissions, which equals the summation of the impacts of PBL height decreases, open burning, and other sources during the winter. The contribution of reduced PBL height on increasing PM_2*.*5_ concentration should be studied to determine the relevant increased PM_2*.*5_ mass. Differences in open burning emissions and other sources during the winter from their emissions during the low concentration period would also be determined to reveal increasing emissions. This required that the reduced mass in PM_2*.*5_ emission assumed that long range transport exhibited no influence. The required reduction in PM_2*.*5_ mass amount should be assigned and distributed to various source sectors with the acceptance of stakeholders. This leads to success in achieving a lower PM_2*.*5_ concentration level in Bangkok than the threshold level.

**Fig. 6:**
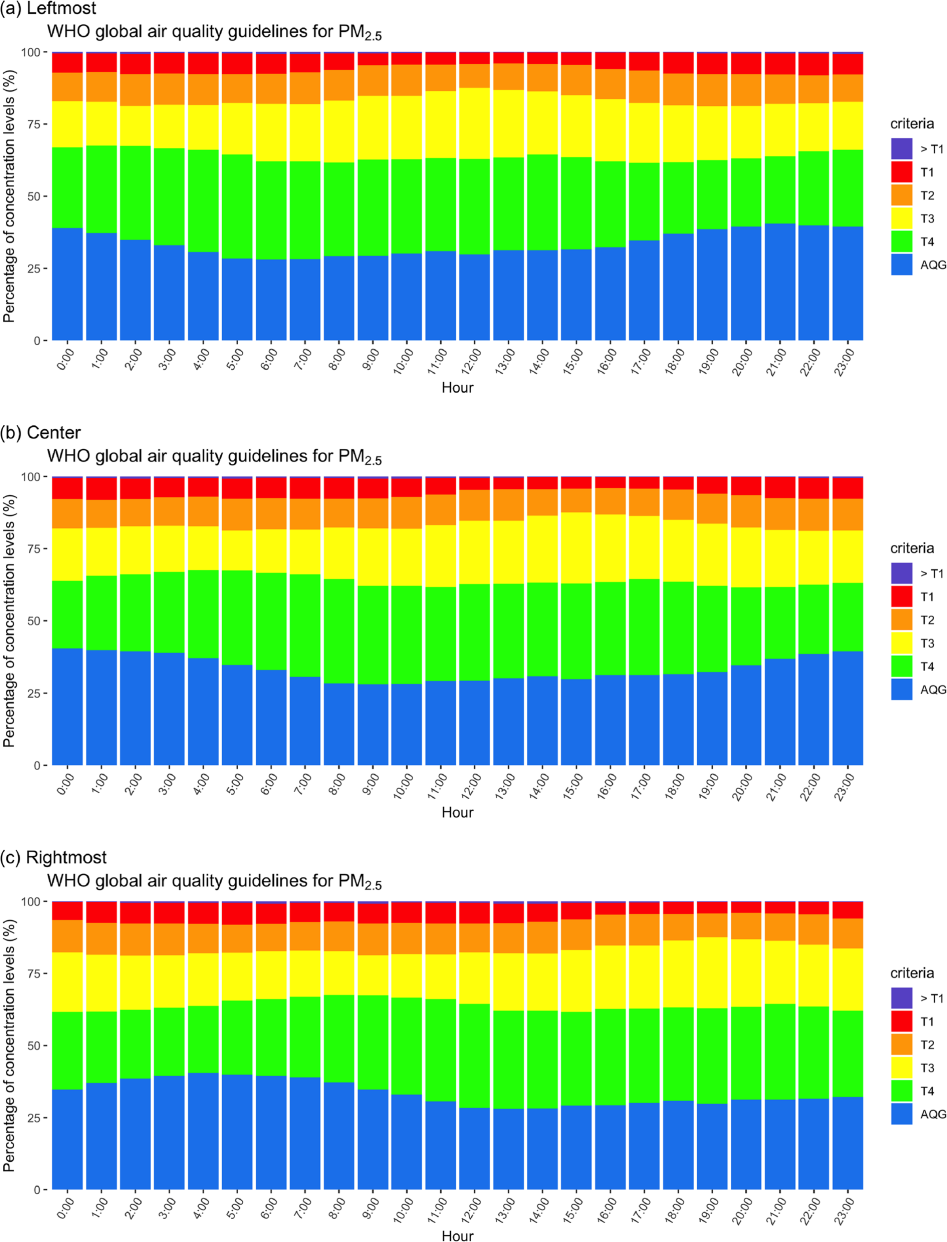
Time variation of PM_2.5_ concentration measured against WHO guideline at station 02 T

Another consideration is investigating the diurnal variation of 24-h moving average PM_2*.*5_ concentration proportion that is associated with each WHO guideline level. First, we investigated the 24-h moving average data recorded at the center. The results are illustrated in Fig. 6b. Blue, green, yellow, orange, red and purple represent percentage of concentrations within AQG value < 15, 15–25, 25–37.5, 37.5–50, 50–75, and ≥ 75 μg m^− 3^, respectively. The proportion of concentrations lower than 15 μg m^− 3^ (blue) was approximately 40% on 0:00 and 26% at 08:00. The contribution during the daytime was quite constant after 08:00 and revealed quite increases during the afternoon to approximately 30%. Then the proportion of concentrations lower than AQG increased again until midnight. These finding imply that air quality in terms of PM_2*.*5_ was safer for health in the night and early morning than in the late morning and afternoon. On the other hand, the proportion of concentration level above the previous Thai NAAQS (red and purple) equaling the interim target 2 (50 μg m^− 3^) was approximately 10%. The smallest proportion presented from 12:00 to 17:00 means that less high concentrations accumulated during the afternoon. This corresponds to a study reporting that high wind speed during the afternoon in Bangkok caused a greater advection process to reduce ambient air PM_2.5_ [37]. Moreover, Thailand changed the national standard from interim target 2 (50 μg m^− 3^) to interim target 3 (37.5 μg m^− 3^). From this result, the exceedances will increase from 10% to approximately 22% (orange, red and purple) but the state of air quality remains at a similar level. Residents may misunderstand and know that the air quality becomes more severe. The government should spend more effort reducing emissions and ambient air concentrations than earlier endeavors.

Using the leftmost and rightmost 24-h moving average PM_2*.*5_ time series in the analysis affected the time shift of the concentration proportions. The proportion of concentrations less than AQG presenting at 5:00 (Fig. 6a), shifted from that occurring at 8:00 (Fig. 6b), using the center 24-h moving average PM_2*.*5_ time series in analysis. This time shift revealed that the events preceding the real occurrence may have resulted in misinterpretation of the analysis. However, it may be useful for some analyses aiming to warn against extreme events. On the other hand, the use of the rightmost 24-h moving average PM_2*.*5_ time series exhibited time-shift lags (Fig. 6c). Presenting a proportion less than that of delayed AQG is the proportion resulting from using the center 24-h moving average occurring at 8:00 moving to 12:00 and the analysis using the rightmost data. The analysis of other stations in Bangkok also presents a time shift (shown in the Supplementary Materials). We suggest that in the analysis using 24-h moving average PM_2*.*5_ data, the position of the stored data should be addressed to avoid misinterpretations and misunderstandings.

## Conclusions

The WHO’s AQG value and interim targets for PM_2*.*5_ in 24-h average, but the continuous ambient air monitoring system provides hourly PM_2*.*5_ time series. Therefore, we converted the hourly PM_2*.*5_ monitoring data to 24-h average time series using the moving average technique, storing the moving average values at the leftmost, center, and rightmost positions. We compared the three 24-h moving time series with hourly PM_2*.*5_ concentration time series in terms of shape (CCF) and magnitude (Euclidean distance). The CCF analysis suggested that all 24-h time series exhibited a marked relationship with hourly PM_2*.*5_ monitoring data. The 24-h moving average concentration recorded at the center was more similar to the hourly concentration time series than the recorded moving average value at the leftmost and rightmost positions. The leftmost and rightmost 24-h moving average time series exhibited the peak of concentration presented before and after the hourly occurring peak with lags from − 13 to − 10 h and 10 to 13 h, respectively. The center 24-h moving average time series had lags from − 2 to 1 h to the hourly time series meaning it showed more similar events to the hourly PM_2*.*5_ fluctuation than the leftmost and rightmost time series. The Euclidean distance to hourly time series were 5.82, 0, and 5.87 μg m^− 3^ for the leftmost, center, and rightmost time series, respectively. The center 24-h moving average time series was more similar to the observed hourly PM_2*.*5_ monitoring data in terms of shape and distance. Thus, comparing with WHO guidelines, values were more suitable than others.

PM_2*.*5_ levels in Bangkok were compared between the center 24-h moving average time series and the AQG values. The observed concentrations were binned in four WHO interim targets and AQG for a 24-h average. The proportion of concentration lower than the AQG level of 15 μg m^− 3^ (blue) was approximately 40% at 0:00 and the portion reduced to 26% at 08:00. The contribution during daytime was more or less contrast after 08:00 increasing slightly to 30% in the afternoon. On the other hand, the proportion of concentration level above the previous Thai NAAQS (red and purple) equaling the interim target 2 (50 μg m^− 3^) was approximately 10%. The smallest proportion of high concentration was observed from 12:00 to 17:00. This implied that the level of PM_2*.*5_ at nighttime was mostly within the interim target 4 (low concentration level). For daytime, the high concentration level (above interim target 3) occurred less from 12:00 to 17:00 meaning less possibility to expose high concentration than that in the morning and late afternoon. Moreover, the Thai national air quality standard of 24-h PM_2*.*5_ was revised from interim target 2 (50 μg m^− 3^) to interim target 3 (37.5 μg m^− 3^). The exceedances will increase from 10 to approximately 22% but the state of air quality remains similar. This may cause residents to misunderstand the information and know the air quality is becoming more severe. The government should spend more effort reducing emissions and ambient air concentrations.

## Supplementary Information

**Additional file 1: Fig. S1.** Correlograms of cross-correlation values between hourly PM_2.5_ concentrations and its 24-h average PM_2.5_ concentrations recorded at leftmost, center, and rightmost, respectively. **Fig. S2.** Diurnal hourly PM_2.5_ proportion against WHO levels for the 03T station. **Fig. S3.** Diurnal hourly PM_2.5_ proportion against WHO levels for the 05T station. **Fig. S4.** Diurnal hourly PM_2.5_ proportion against WHO levels for the 10T station. **Fig. S5.** Diurnal hourly PM_2.5_ proportion against WHO levels for the 11T station. **Fig. S6.** Diurnal hourly PM_2.5_ proportion against WHO levels for the 12T station. **Fig. S7.** Diurnal hourly PM_2.5_ proportion against WHO levels for the 50T station. **Fig. S8.** Diurnal hourly PM_2.5_ proportion against WHO levels for the 52T station. **Fig. S9.** Diurnal hourly PM_2.5_ proportion against WHO levels for the 53T station. **Fig. S10.** Diurnal hourly PM_2.5_ proportion against WHO levels for the 54T station. **Fig. S11.** Diurnal hourly PM_2.5_ proportion against WHO levels for the 59T station. **Fig. S12.** Diurnal hourly PM_2.5_ proportion against WHO levels for the 61T station.

## Data Availability

All data generated or analyzed during this study are included within the submitted manuscript and the Supplementary Materials.
