# Supplementary material for: Assessment of 24-h moving average PM2.5 concentrations in Bangkok, Thailand against WHO guidelines
Source: Sustain Environ Res. 2023 Jan 24;33(1):3. doi: 10.1186/s42834-023-00165-y (PMC9872734; doi:10.1186/s42834-023-00165-y)
Supplement: Supplementary file 1 — Additional file 1: Fig. S1. Correlograms of cross-correlation values between hourly PM2.5 concentrations and its 24-h average PM2.5 concentrations recorded at leftmost, center, and rightmost, respectively. Fig. S2. Diurnal hourly PM2.5 proportion against WHO levels for the 03T station. Fig. S3. Diurnal hourly PM2.5 proportion against WHO levels for the 05T station. Fig. S4. Diurnal hourly PM2.5 proportion against WHO levels for the 10T station. Fig. S5. Diurnal hourly PM2.5 proportion against WHO levels for the 11T station. Fig. S6. Diurnal hourly PM2.5 proportion against WHO levels for the 12T station. Fig. S7. Diurnal hourly PM2.5 proportion against WHO levels for the 50T station. Fig. S8. Diurnal hourly PM2.5 proportion against WHO levels for the 52T station. Fig. S9. Diurnal hourly PM2.5 proportion against WHO levels for the 53T station. Fig. S10. Diurnal hourly PM2.5 proportion against WHO levels for the 54T station. Fig. S11. Diurnal hourly PM2.5 proportion against WHO levels for the 59T station. Fig. S12. Diurnal hourly PM2.5 proportion against WHO levels for the 61T station. [file 42834_2023_165_MOESM1_ESM.docx]

**Supplementary material**


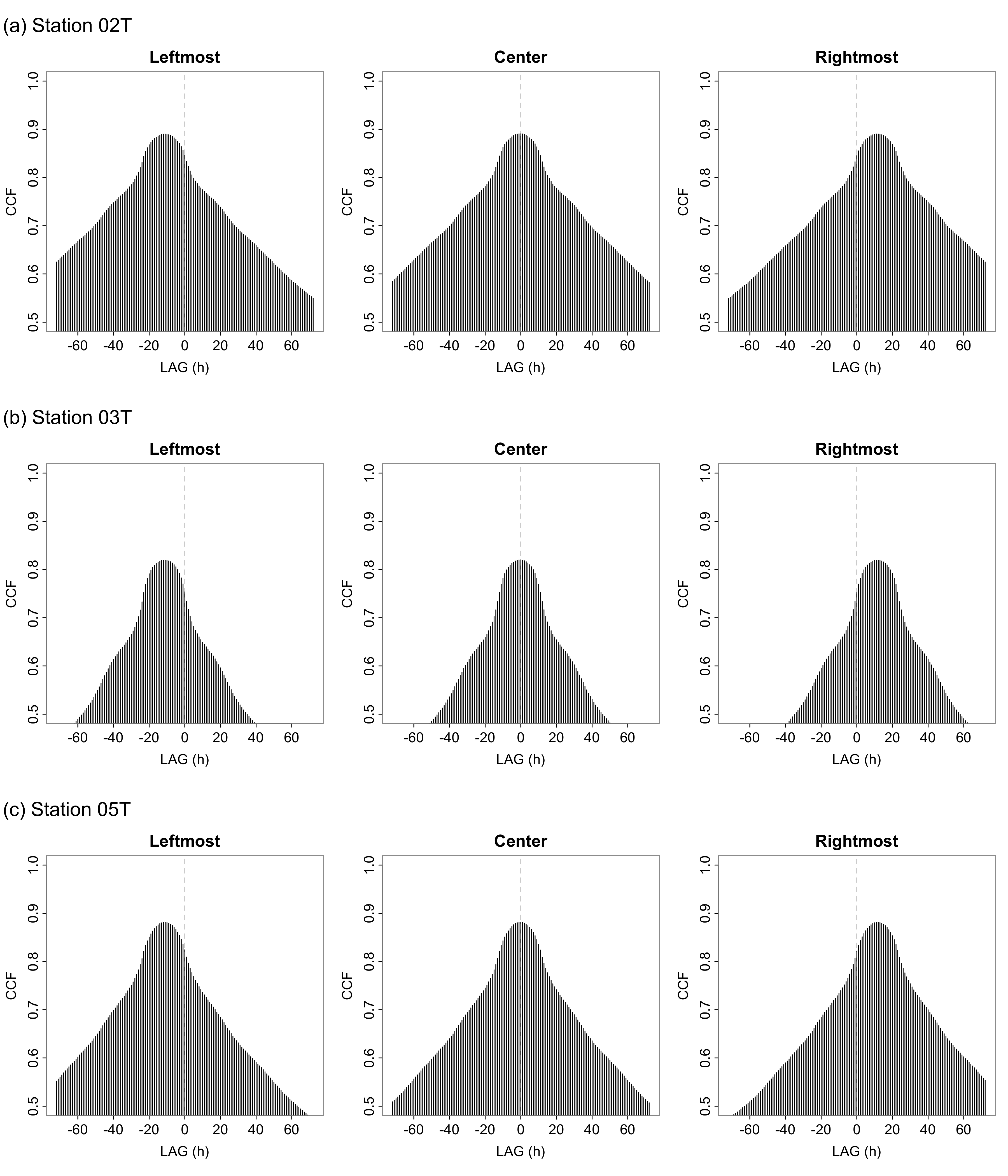


**Fig. S1** Correlograms of cross-correlation values between hourly PM_2.5_ concentrations and its 24-h average PM_2.5_ concentrations recorded at leftmost, center, and rightmost, respectively


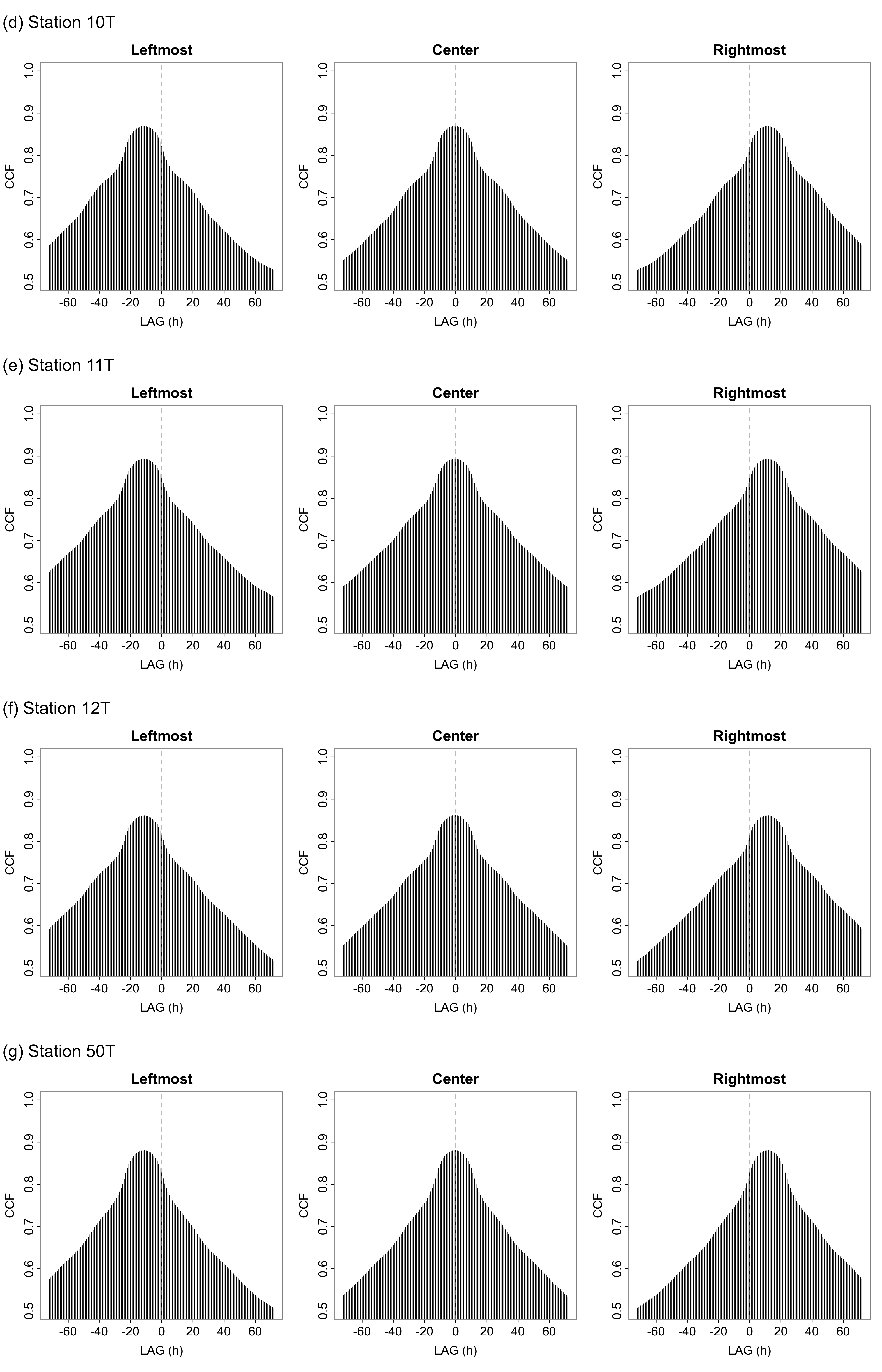


**Fig. S1** Correlograms of cross-correlation values ... (Cont.)


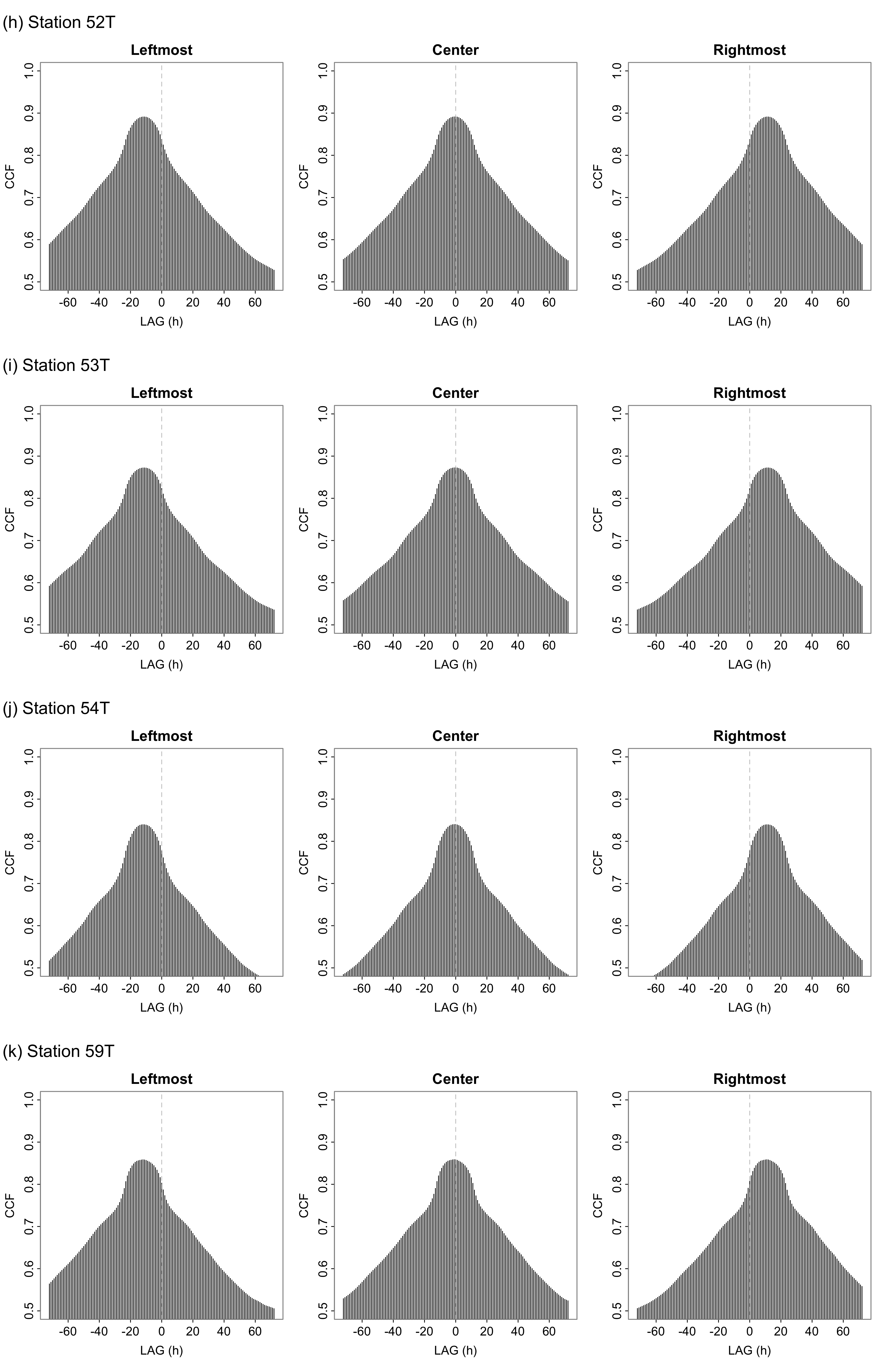


**Fig. S1** Correlograms of cross-correlation values ... (Cont.)


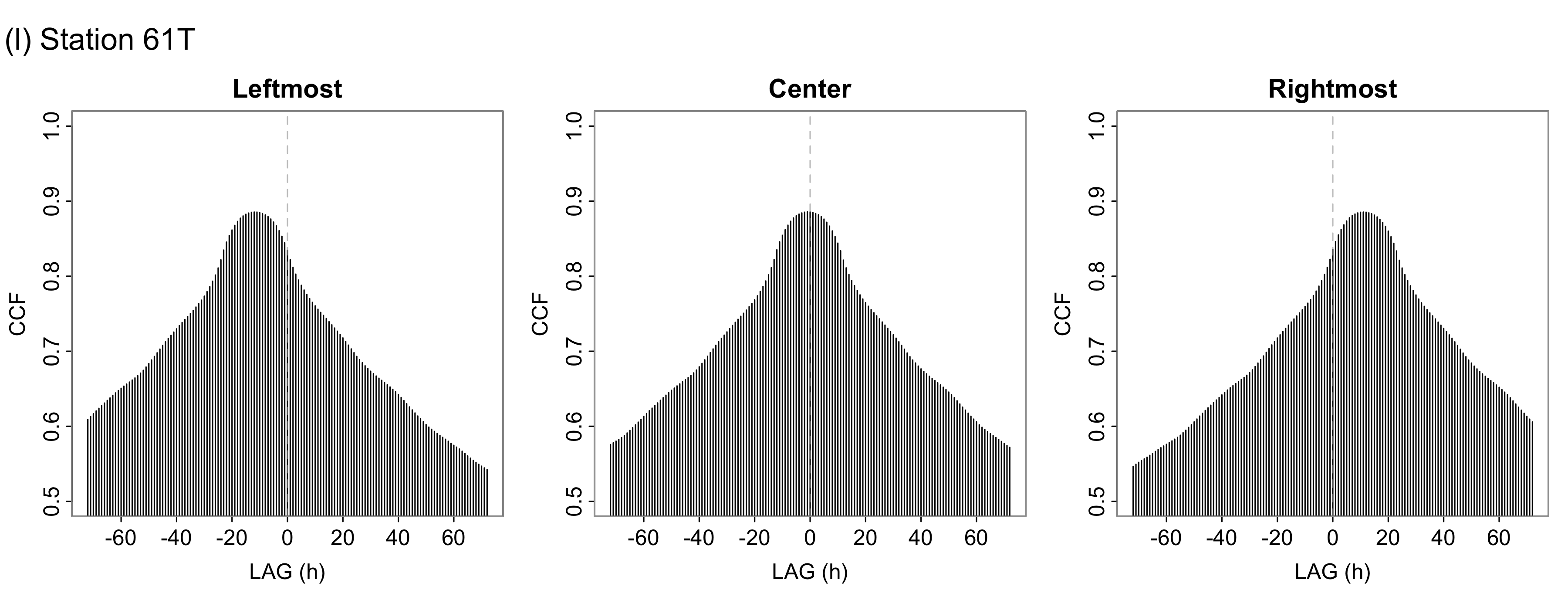


**Fig. S1** Correlograms of cross-correlation values ... (Cont.)


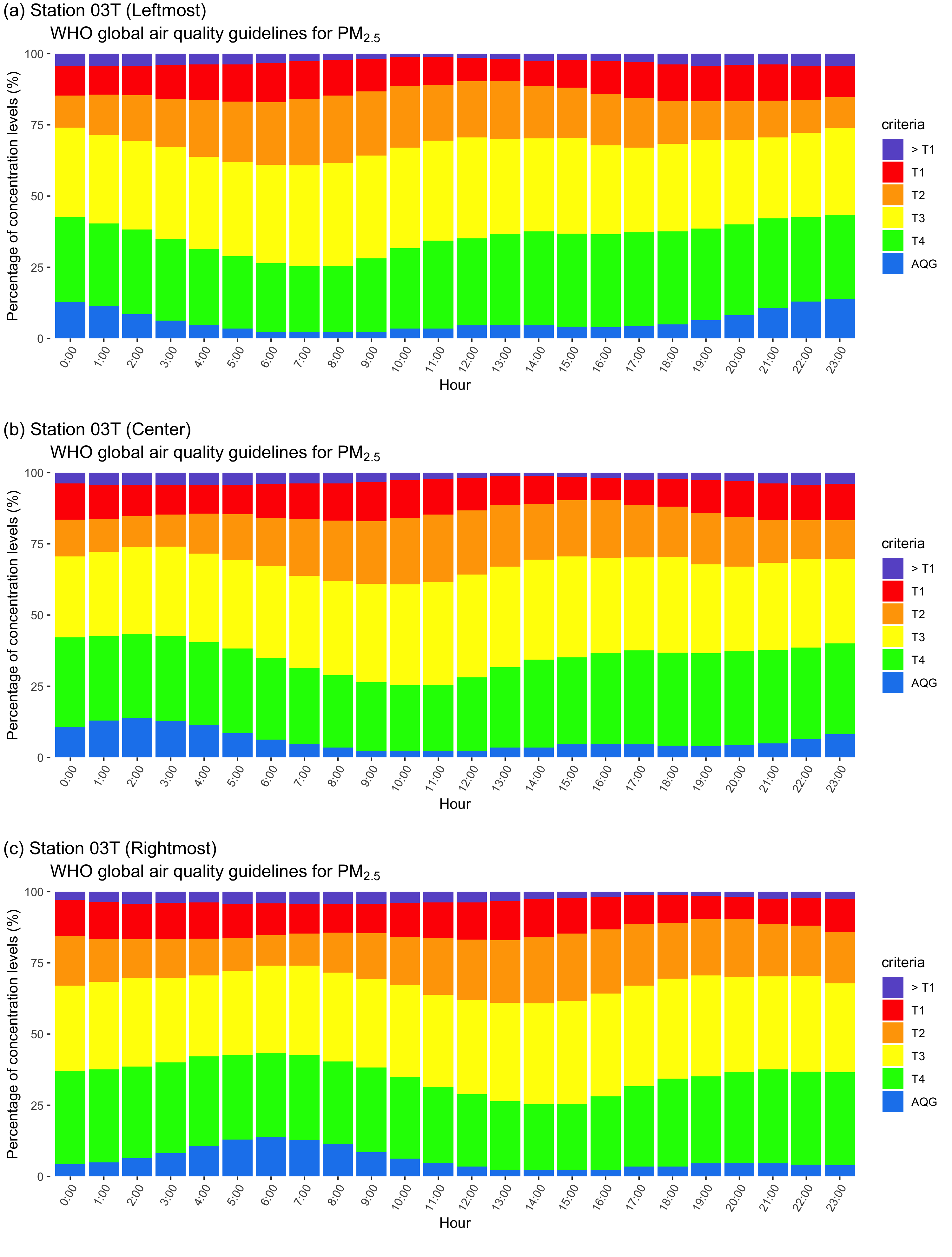


**Fig. S2** Diurnal hourly PM_2.5_ proportion against WHO levels for the 03T station

**
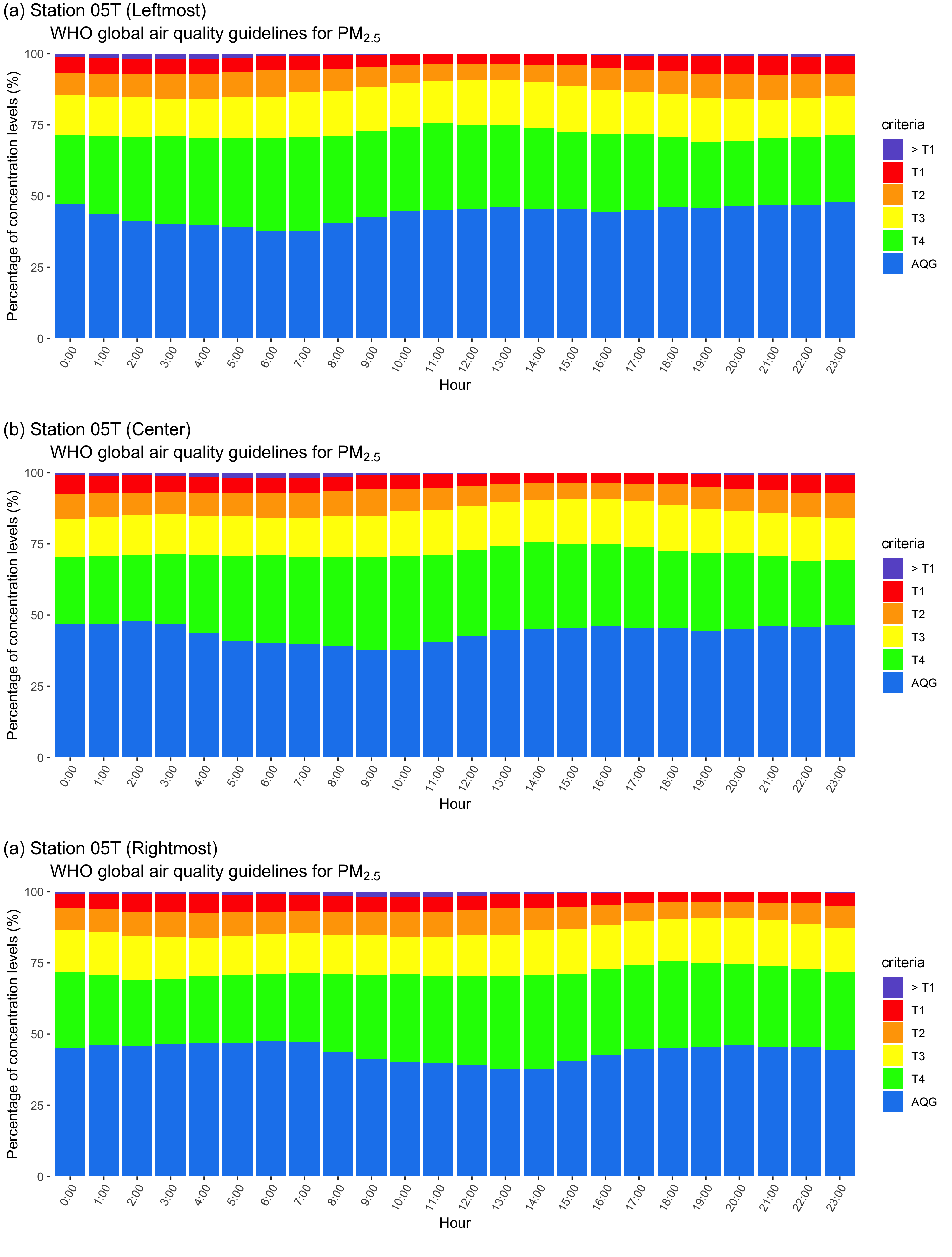
**

**Fig. S3** Diurnal hourly PM_2.5_ proportion against WHO levels for the 05T station


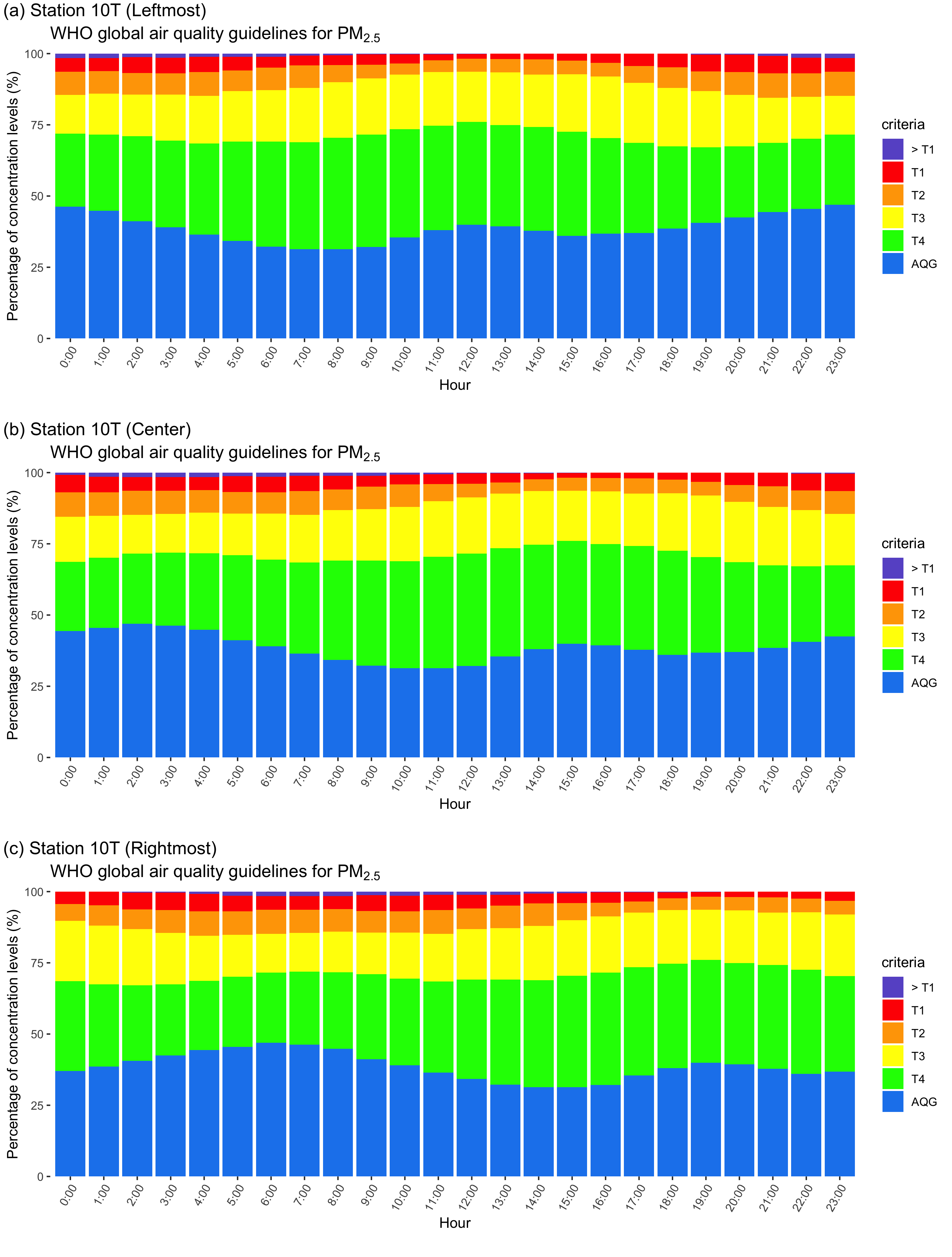


**Fig. S4** Diurnal hourly PM_2.5_ proportion against WHO levels for the 10T station


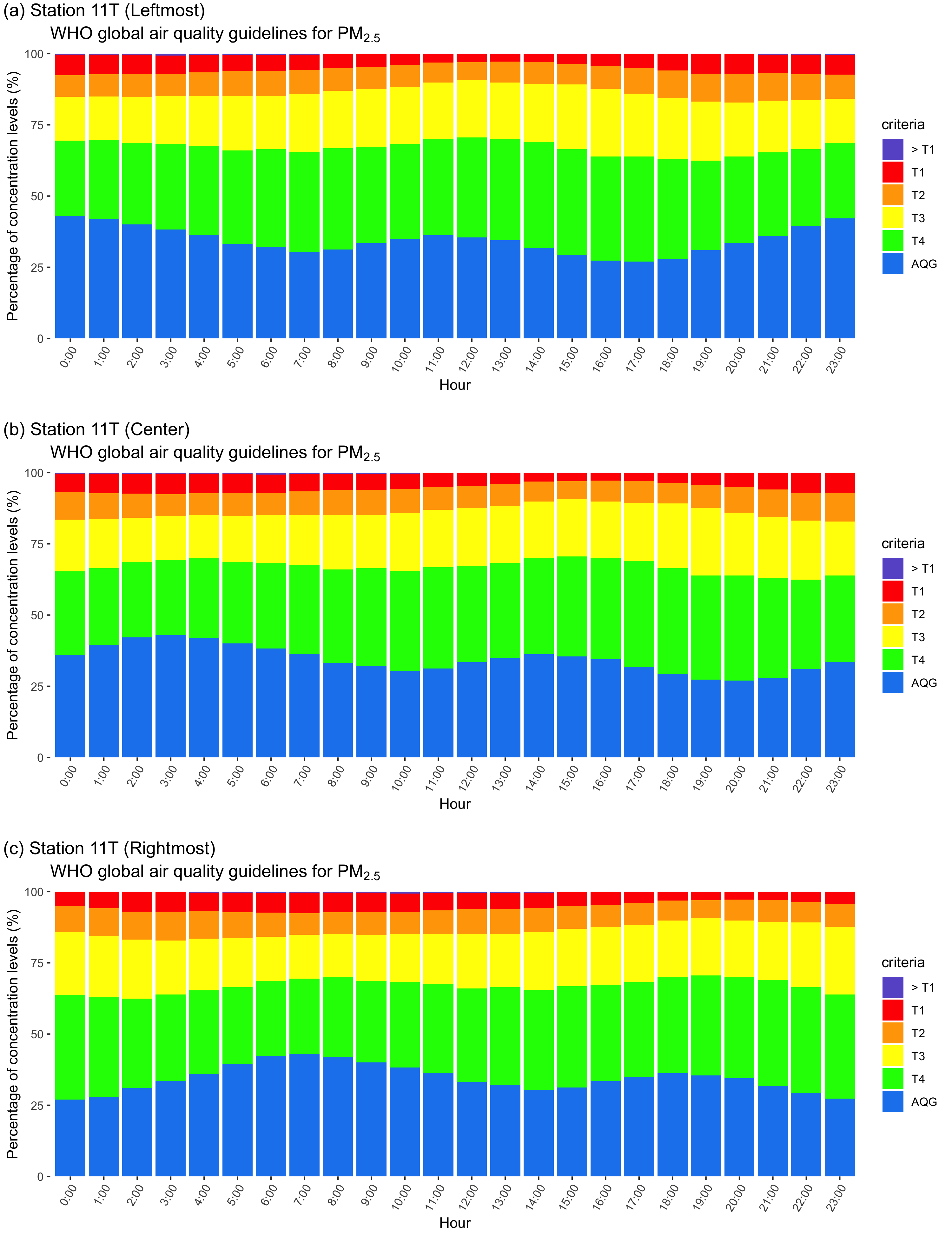


**Fig. S5** Diurnal hourly PM_2.5_ proportion against WHO levels for the 11T station


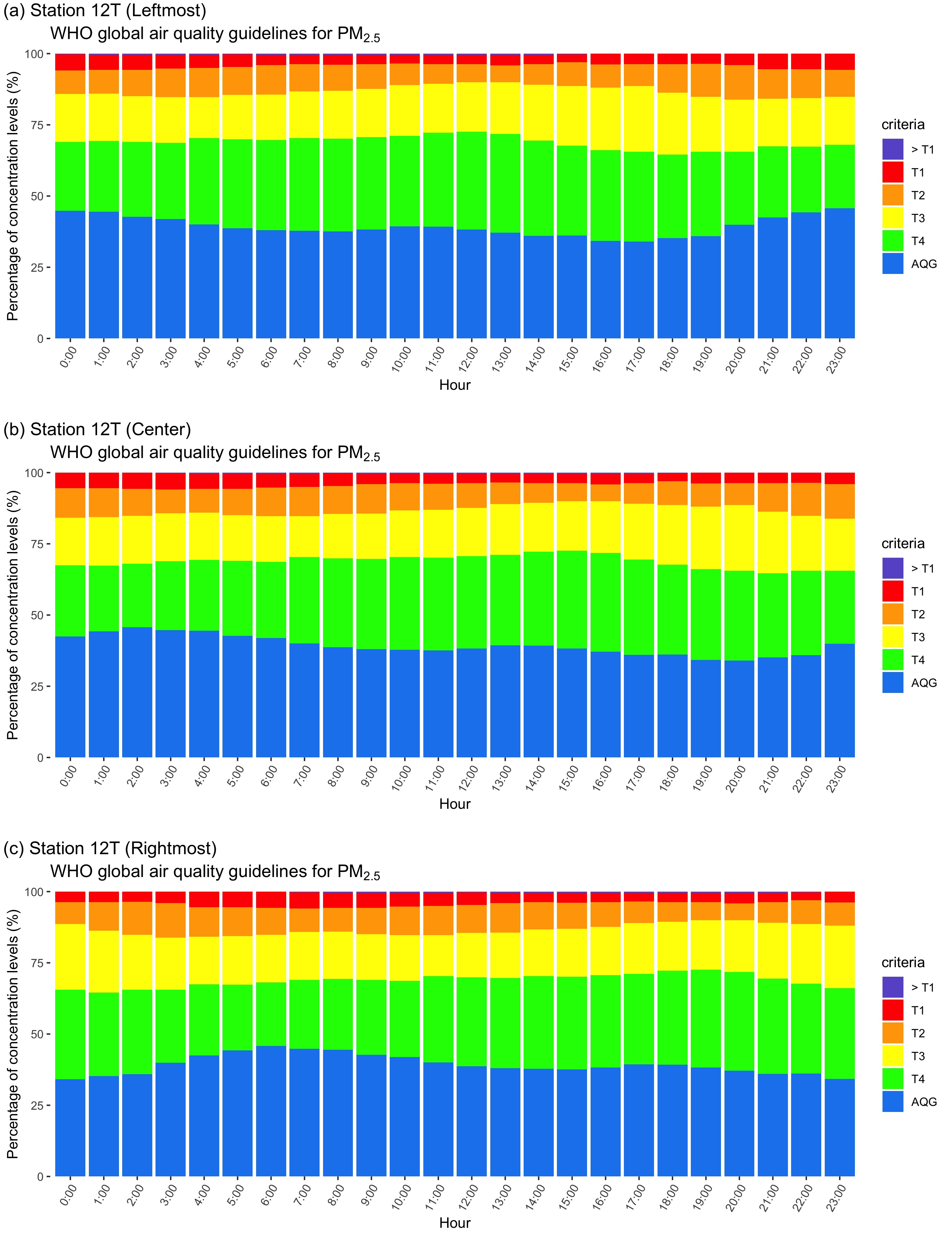


**Fig. S6** Diurnal hourly PM_2.5_ proportion against WHO levels for the 12T station


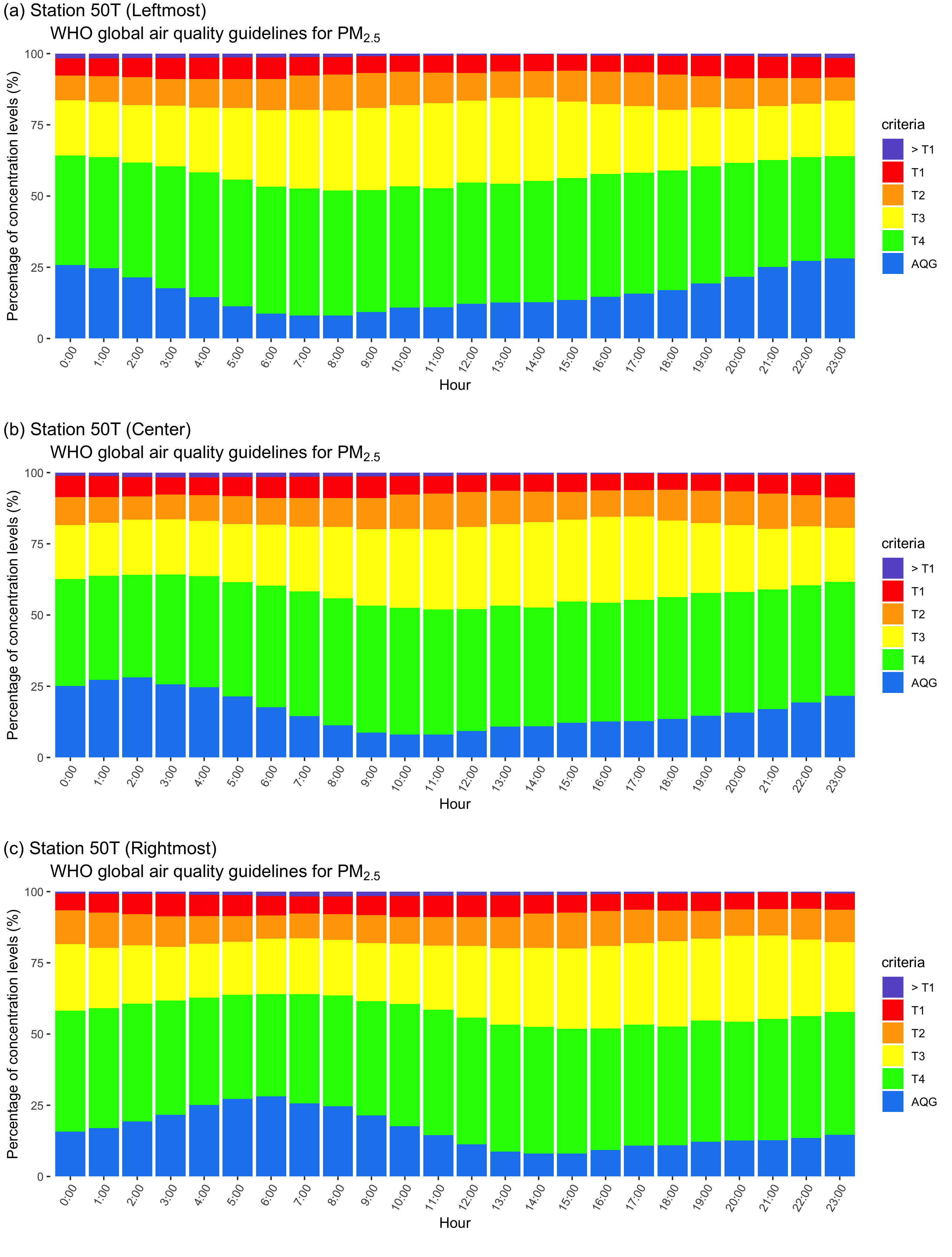


**Fig. S7** Diurnal hourly PM_2.5_ proportion against WHO levels for the 50T station


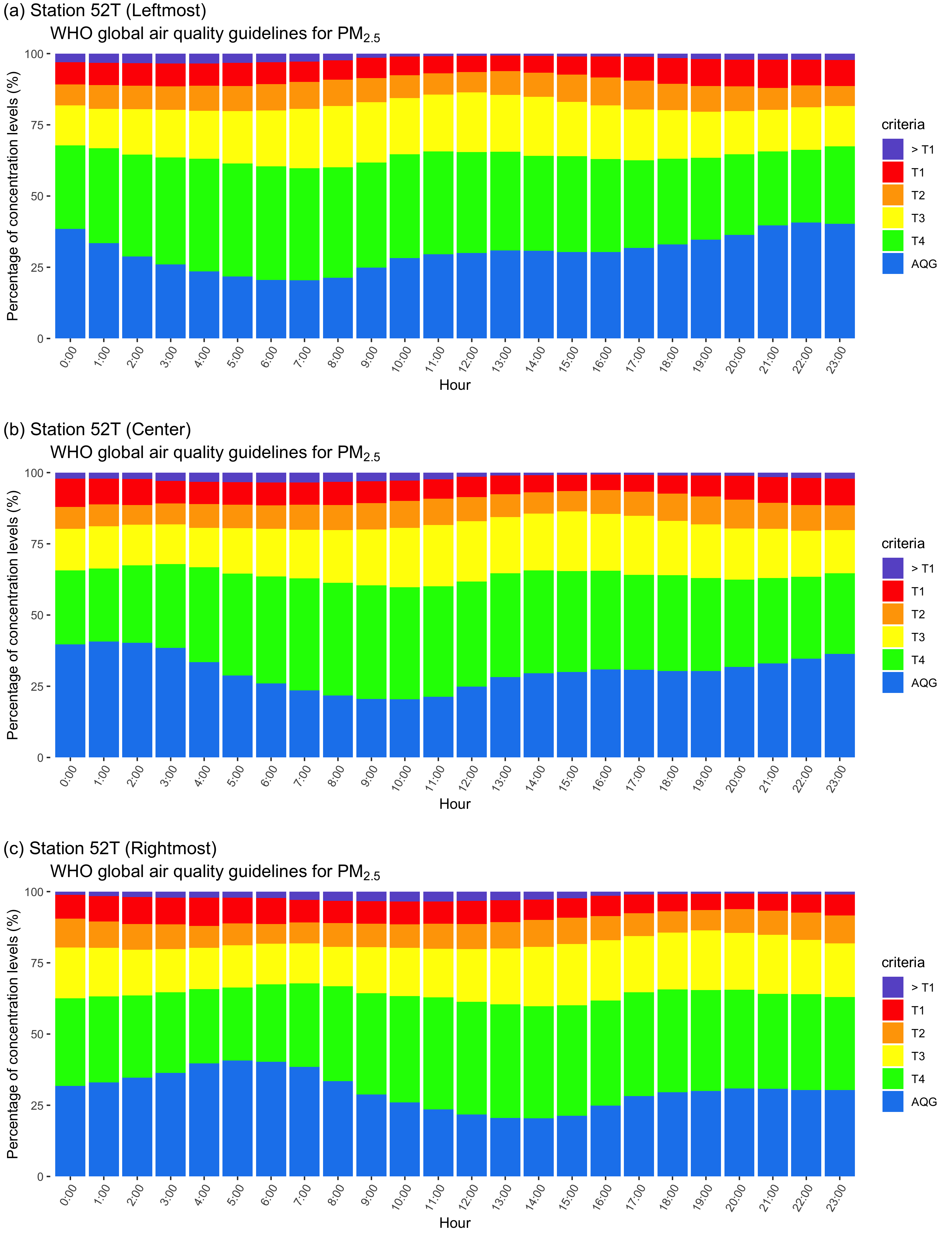


**Fig. S8** Diurnal hourly PM_2.5_ proportion against WHO levels for the 52T station


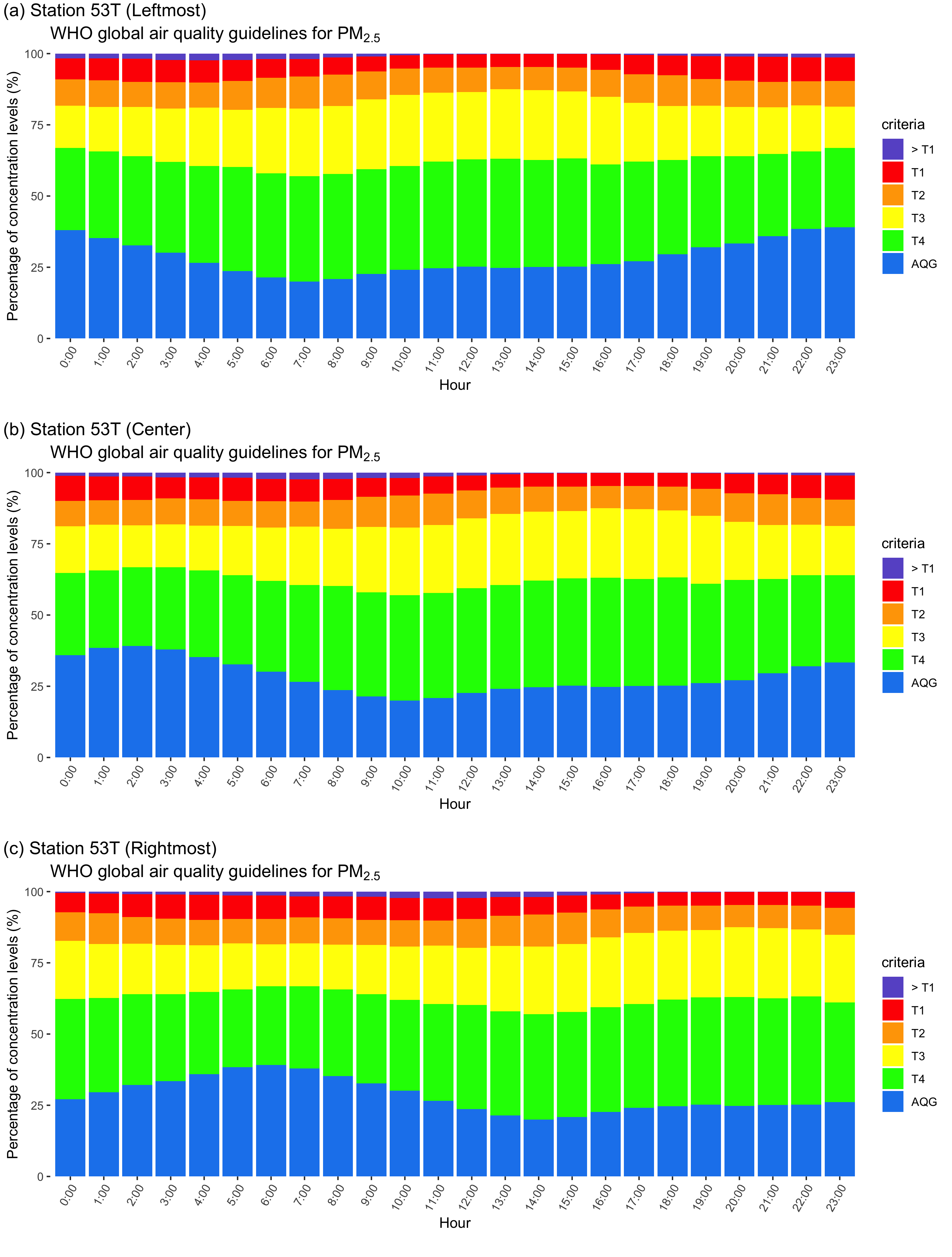


**Fig. S9** Diurnal hourly PM_2.5_ proportion against WHO levels for the 53T station


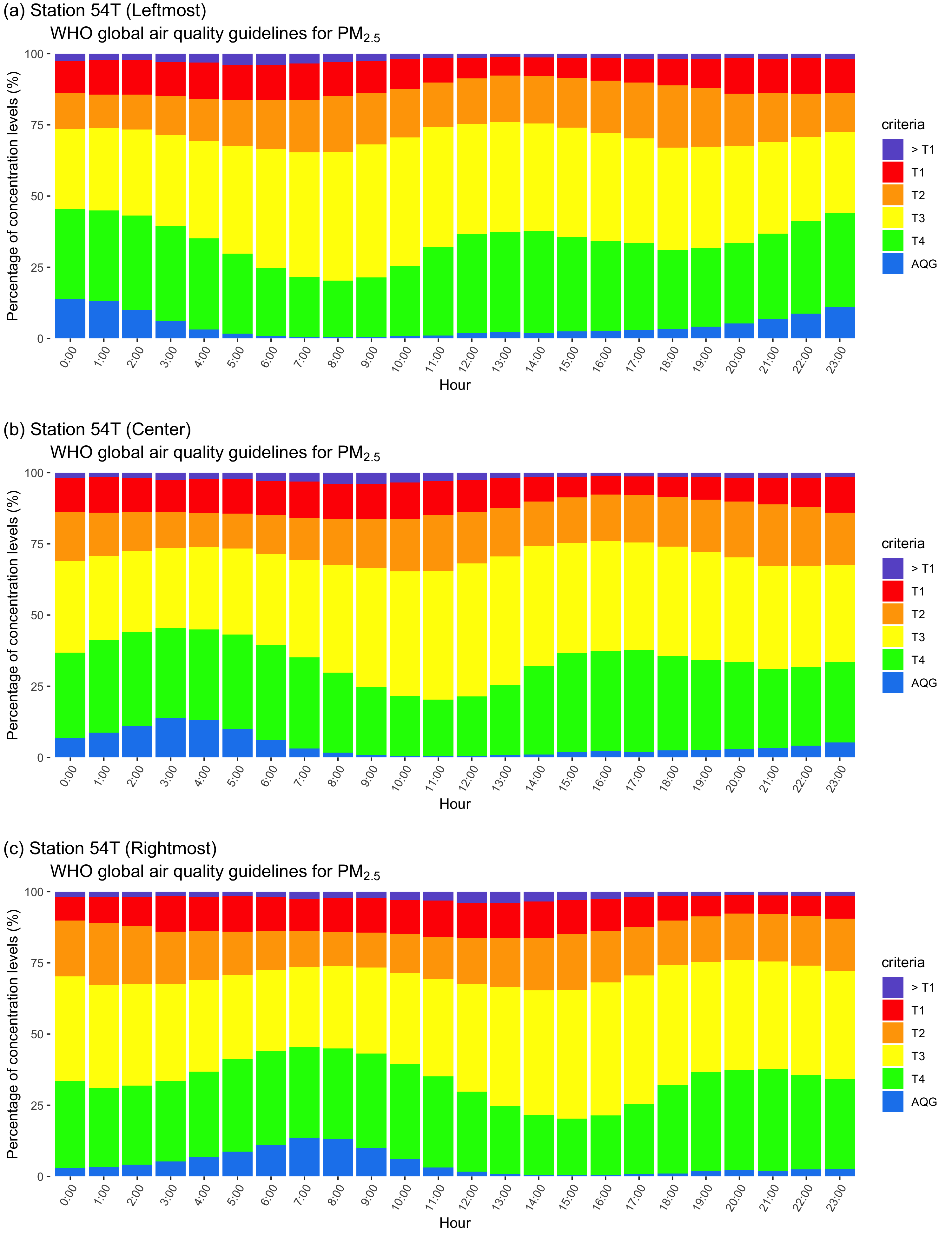


**Fig. S10** Diurnal hourly PM_2.5_ proportion against WHO levels for the 54T station


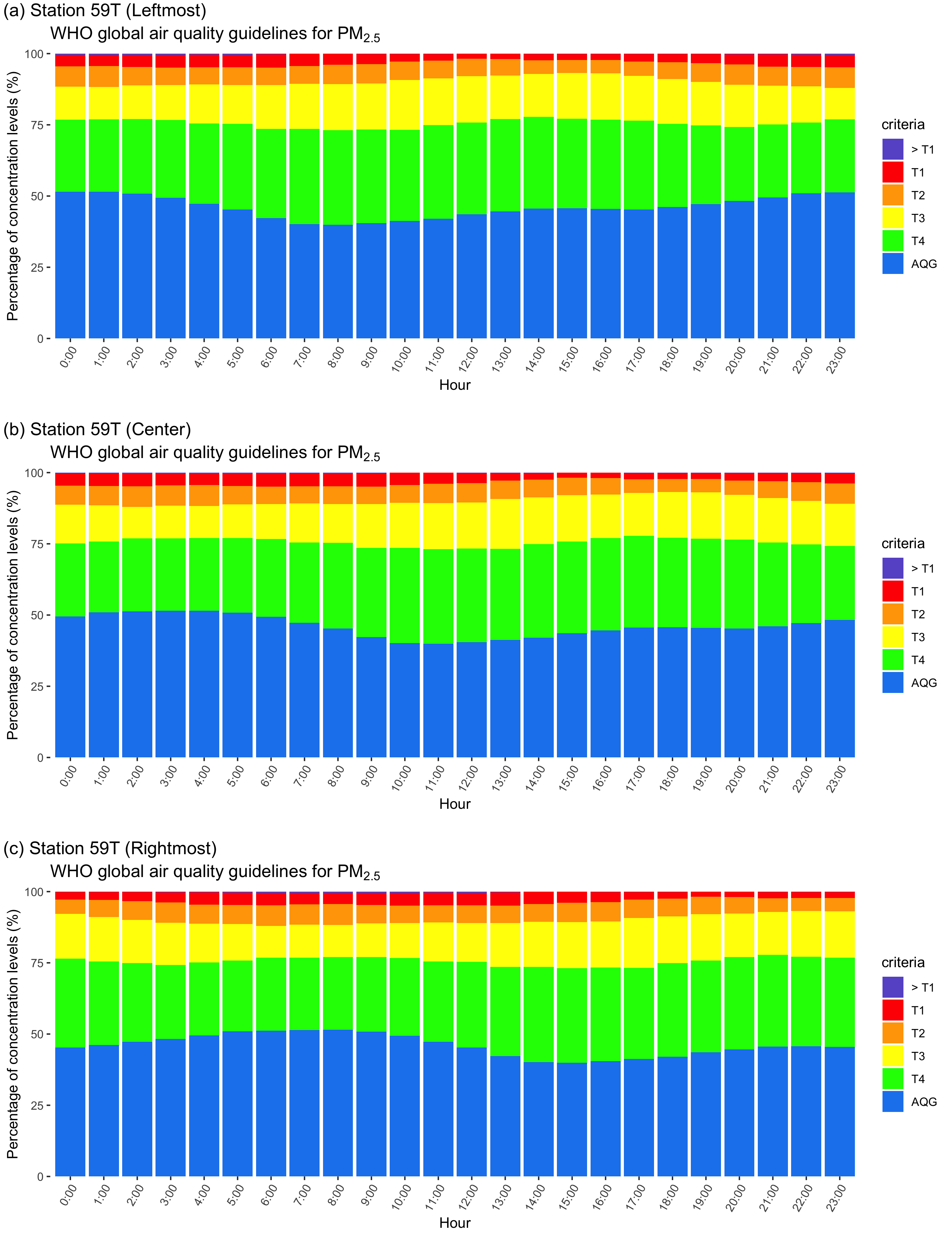


**Fig. S11** Diurnal hourly PM_2.5_ proportion against WHO levels for the 59T station


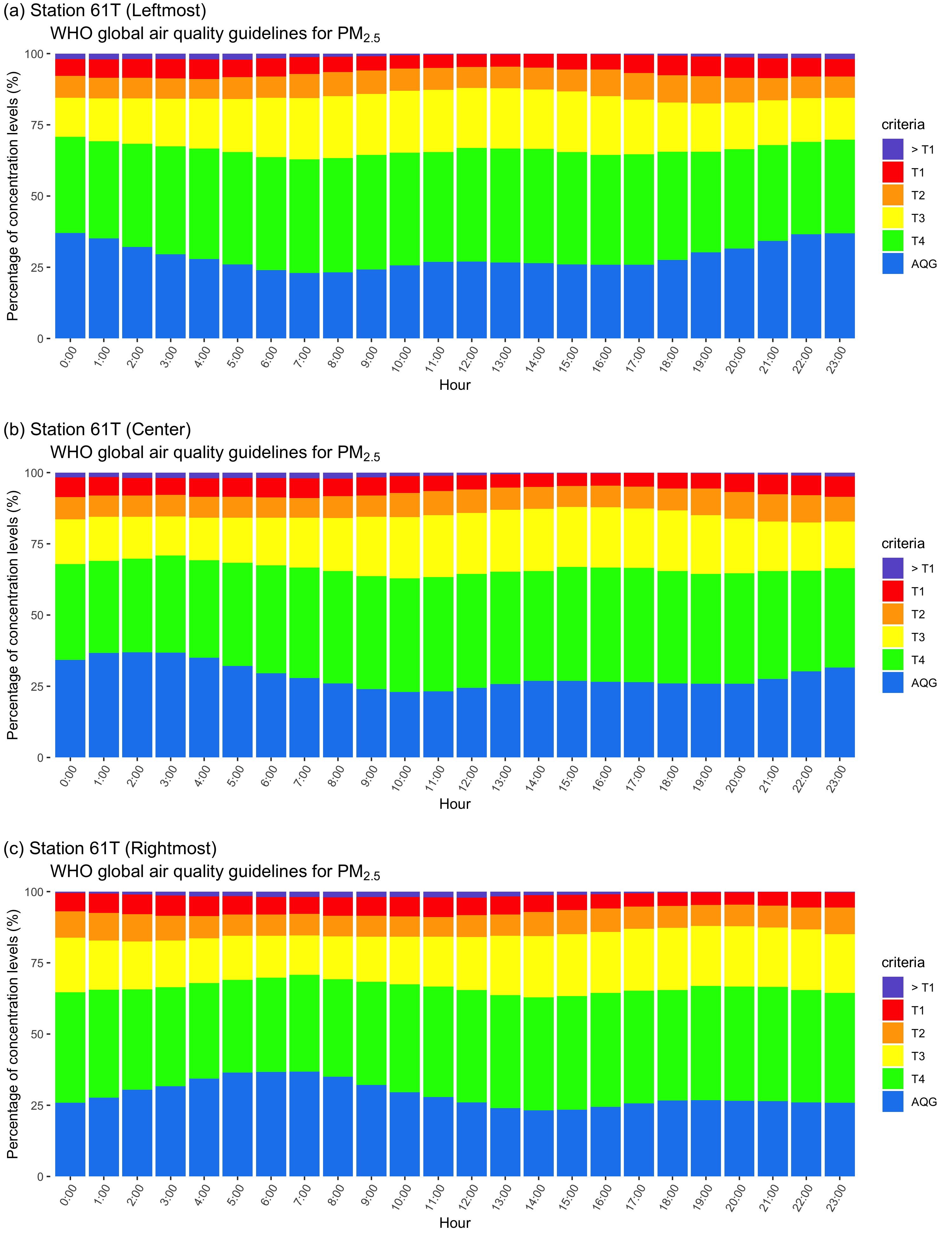


**Fig. S12** Diurnal hourly PM_2.5_ proportion against WHO levels for the 61T station
